# Structure-Function of the Tumor Suppressor BRCA1

**DOI:** 10.5936/csbj.201204005

**Published:** 2012-03-19

**Authors:** Serena L. Clark, Ana M. Rodriguez, Russell R. Snyder, Gary D.V. Hankins, Darren Boehning

**Affiliations:** a Department of Neuroscience and Cell Biology, University of Texas Medical Branch, Galveston, TX, 77550; b Department of Obstetrics and Gynecology, University of Texas Medical Branch, Galveston, TX, 77550; c Sealy Center for Structural Biology and Molecular Biophysics, University of Texas Medical Branch, Galveston, TX, 77550; † These authors contributed equally

## Abstract

BRCA1, a multi-domain protein, is mutated in a large percentage of hereditary breast and ovarian cancers. BRCA1 is most often mutated in three domains or regions: the N-terminal RING domain, exons 11-13, and the BRCT domain. The BRCA1 RING domain is responsible for the E3 ubiquitin ligase activity of BRCA1 and mediates interactions between BRCA1 and other proteins. BRCA1 ubiquitinates several proteins with various functions. The BRCA1 BRCT domain binds to phosphoproteins with specific sequences recognized by both BRCA1 and ATM/ATR kinases. Structural studies of the RING and BRCT domains have revealed the molecular basis by which cancer causing mutations impact the functions of BRCA1. While no structural data is available for the amino acids encoded by exons 11-13, multiple binding sites and functional domains exist in this region. Many mutations in exons 11-13 have deleterious effects on the function of these domains. In this mini-review, we examine the structure-function relationships of the BRCA1 protein and the relevance to cancer progression.

## Clinical significance of BRCA1 in breast and ovarian cancers

Hereditary Breast and Ovarian Cancer (HBOC) is a syndrome resulting in an increased lifetime risk for developing breast and/or ovarian cancer. The genetic basis of HBOC is usually an inherited germline mutation in one allele of either the *BRCA1* or *BRCA2* genes and subsequent loss of heterozygosity in somatic tissues [1]. Some of the trademarks of this syndrome include multiple family members with breast and/or ovarian cancer, personal history of both breast and ovarian cancer, development of breast or ovarian cancer at an early age, and family or personal history of male breast cancer [1].

Mutations in *BRCA1* and *BRCA2* are responsible for the majority of HBOC cases [1]. According to the literature, 10% of ovarian cancer cases and 3–5% of breast cancer cases are associated with *BRCA1* or *BRCA2* mutations [1]. In the presence of a *BRCA1* mutation, women have a 70-80% lifetime risk of developing breast cancer and a 50% risk of developing ovarian cancer. Women carrying a *BRCA2* mutation have a 50-60% lifetime risk of developing breast cancer and a 30% risk of developing ovarian cancer [2]. These genes belong to the tumor suppressor gene family for their capacity to repair damaged DNA through a process known as DNA double-strand break repair [3]. Therefore, an inherited mutation in either of these genes combined with loss of heterozygosity predisposes cells to chromosomal instability and greatly increases the probability of malignant transformation and cancer development. Interestingly, multiple other potential functions have been proposed for the BRCA1 and BRCA2 proteins that may have an impact on their tumor suppressor function [4].

The management of HBOC syndrome is an evolving area, and clearly much more research is needed to understand the molecular basis of cancer progression in these patients. The linkage of *BRCA1* and *BRCA2* to early-onset hereditary breast cancer was discovered in 1990 and 1994 respectively [5, 6]. Since then, BRCA genotyping is now used to determine patient counseling, management decisions, and prognosis of this syndrome [7]. However, inconsistent and limited data exist regarding the clinical course of BRCA-mutated patients after cancer develops [8]. A published meta-analysis for *BRCA1*-related tumors reported a worse outcome among the breast cancer patients carrying a mutated BRCA gene [7], while *BRCA1* mutated ovarian cancer patients had a more favorable clinical outcome [9]. Other studies have reported that both *BRCA1*-mutated breast and ovarian tumors have a better outcome [8, 10]. This is likely due to increased sensitivity of BRCA mutated cells to chemotherapeutics targeting DNA such as anti-metabolites, alkylating agents, and topoisomerase inhibitors [11]. However, more research into the molecular basis by which the BRCA proteins function as tumor suppressors and the clinical significance is clearly needed.

**Figure 1:**
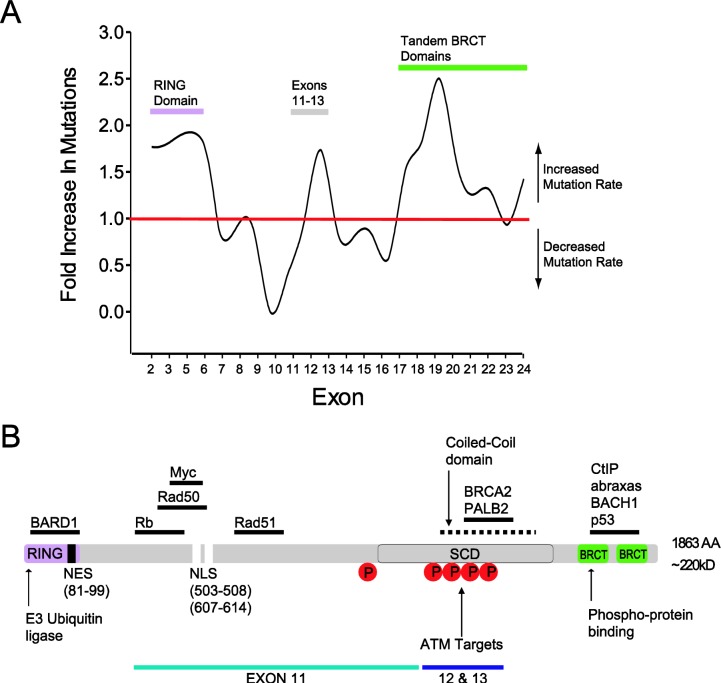
**BRCA1 mutations occur at the highest rates in the RING domain, exons 11-13 and the BRCT domain**. A) BRCA1 clinically relevant mutations from the Breast Cancer Information Core (BIC). Fold increase in mutations were calculated as mutations per codon length of each exon/total mutations per total BRCA1 codons. 1.0 on the y-axis indicates the total average mutations per codon for BRCA1. Corresponding domains are indicated above the graph. B) Domain map of BRCA1. RING, serine containing domain (SCD), and BRCT domains are indicated. NES and NLS sequences are also depicted. Horizontal solid black lines indicate protein binding domains for the listed binding partners. Red circles mark phosphorylation sites.

Over 1700 unique *BRCA1* mutations have been reported to the Breast Cancer Information Core Database [12]. Of these mutations, 858 have been confirmed as being “clinically significant.” Clinically significant mutations cause an increased risk of cancer and result in a protein with reduced function or no protein product. Three domains of the BRCA1 protein are mutated in cancer patients with relatively high frequency. These domains include the RING domain (exons 2-7), a region encoded by exons 11-13, and the BRCT domain (exons16-24) (Figure 1). The RING domain functions as an E3 ubiquitin ligase. The amino acids encoded by exons 11-13 contain protein binding domains for a number of diverse proteins. The BRCT domain is a phosphoprotein binding domain with specificity for proteins phosphorylated by ATM/ATR kinases

Understanding the structural biology of BRCA1 and BRCA2 is important for elucidating both physiologic and pathophysiologic function of these proteins. As shown in Table 1, multiple structures have been solved for the BRCA1 RING and BRCT domains and associated proteins, including clinically relevant mutants.

**Table 1:** Summary of published BRCA1 structures.

Domain	Method	Description	PDB ID	Ref.
RING	NMR	BRCA1/BARD1 RING-domain heterodimer	1JM7	[13]
BRCT	X-Ray	BRCA1 BRCT repeat region	IJNX	[14]
BRCT	X-Ray	BRCA1 BRCT mutation M1775R	1N5O	[15]
BRCT	X-Ray	BRCA1 BRCT mutant M1775K	2ING	[16]
BRCT	NMR	BRCA1 BRCT-c domain	1OQA	[17]
BRCT+peptide	X-Ray	BRCA1 BRCT with BACH1 phosphopeptide	1T29	[18]
BRCT+peptide	X-Ray	BRCA1 BRCT with BACH1 phosphopeptide	1T15	[19]
BRCT+peptide	X-Ray	BRCA1 BRCT with CtIP phosphopeptide	1Y98	[16]
BRCT+peptide	X-Ray	BRCA BRCT with Acetyl-CoA Carboxylase 1 phosphopeptide	3COJ	[20]
BRCT+peptide	X-Ray	BRCA1 BRCT with phosphopeptide	1T2V	[15]
BRCT+peptide	X-Ray	BRCA1 BRCT V1809F with phosphopeptide	1T2U	[15]
BRCT+peptide	X-Ray	BRCA1 BRCT with a minimal recognition tetrapeptide ( amidated C-terminus)	3K0H	[21]
BRCT+peptide	X-Ray	BRCA1 BRCT with a minimal recognition tetrapeptide (free carboxy C-terminus)	3K0K	[21]
BRCT+peptide	X-Ray	BRCA1 BRCT D1840T with a minimal recognition tetrapeptide (amidated C-terminus)	3K15	[21]
BRCT+peptide	X-Ray	BRCA1 BRCT D1840T with a minimal recognition tetrapeptide ( free carboxy C-terminus)	3K16	[21]
BRCT+peptide	X-Ray	BRCA1 BRCT G1655D with phosphopeptide	3PXA	[22]
BRCT+peptide	X-Ray	BRCA1 BRCT T1700A with phosphopeptide	3PXB	[22]
BRCT+peptide	X-Ray	BRCA1 BRCT R1699Q with phosphopeptide	3PXC	[22]
BRCT+peptide	X-Ray	BRCA1 BRCT R1835P with phosphopeptide	3PXD	[22]
BRCT+peptide	X-Ray	BRCA1 BRCT E1836K with phosphopeptide	3PXE	[22]

In this review we will focus on the structural basis by which the BRCA1 protein functions as a tumor suppressor, and highlight the importance of these studies to understanding the pathophysiology and clinical outcomes of breast and ovarian cancers.

## RING domain

**Figure 2:**
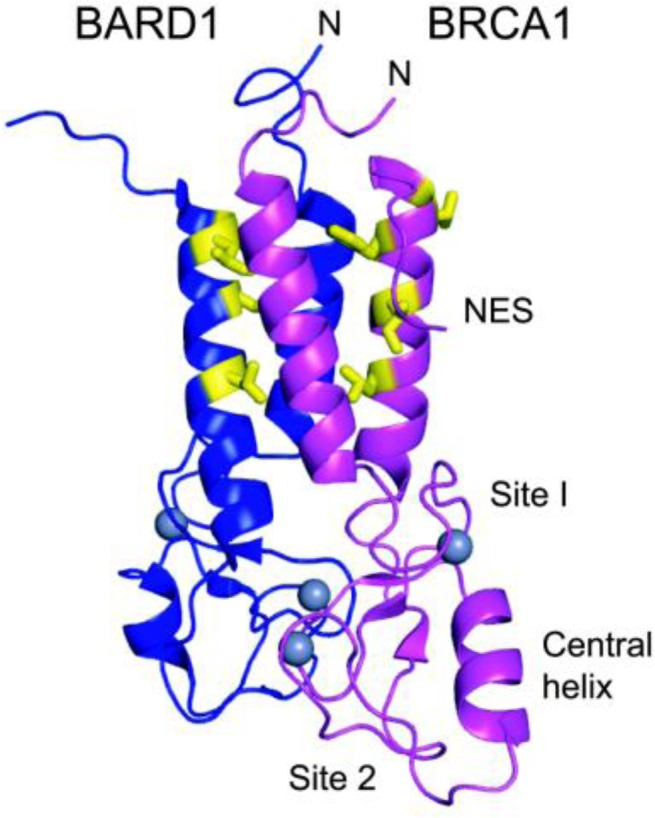
**BRCA1 RING domain**. The RING domain contains a RING finger and two flanking alpha helices. The RING finger consists of a core of β-strands, a central helix, and two Zn^2+^ binding sites. BRCA1 (pink) forms a heterodimer with the RING domain of BARD1 (blue). Critical NES residues are highlighted in yellow. N-termini of each strand are labeled. Structural model is derived from PDB accession number 1JM7 and rendered using POLYVIEW-3D [31].

The RING (Really Interesting New Gene) domain of BRCA1 consists of a RING finger and two flanking alpha helices encompassing amino acids 1-109 (exons 2-7) [13, 23]. Through seven conserved cysteine residues and one conserved histidine residue, the RING finger coordinates two Zn^2+^ atoms which stabilize the RING structure [24, 25]. The RING finger forms a globular structure with a core three strand β-sheet and a central helix, while the flanking helices align perpendicular to the RING finger (Figure 2). The RING finger, which is a highly conserved domain found in a large number of proteins, is responsible for the E3-ubiquitin ligase activity of BRCA1 [26]. The N and C-terminal helices are responsible for the interaction of BRCA1 with BARD1 (BRCA1 Associated RING Domain protein 1), a major BRCA1 binding partner that also contains a RING domain [27]. The ubiquitin ligase activity of BRCA1 is dramatically increased by formation of the BRCA1/BARD1 heterodimer [28]. As with all E3-ubiquitin ligases, ubiquitination of a substrate can only occur through interaction with an E2 ubiquitin-conjugating enzyme. UbcH5, as well as other E2 enzymes, binds to the surface of BRCA1 opposite the binding interface with BARD1 [29]. The large number of cancer predisposing mutations that affect the interaction of BRCA1/BARD1 or BRCA1/UbcH5 as well as the RING E3 ligase function suggest that the ubiquitin ligase activity of BRCA1 is essential for its tumor suppressor function (but see [30]).

### Structure-Function of the RING domain

We have gained the most information about the structure-function of the BRCA1 RING domain from the structure of the BRCA1/BARD1 heterodimer. BARD1 also contains a RING domain with sequence and structural homology to BRCA1, including two flanking alpha helices. The N-terminal alpha-helix of BRCA1 aligns in an antiparallel fashion with the C-terminal alpha helix of BARD1. Conversely, the C-terminal alpha-helix of BRCA1 is antiparallel to the N-terminal alpha-helix of BARD1 (Figure 2) [13]. The four helix bundle creates a large buried hydrophobic region and stabilizes the heterodimer, while interactions between the BRCA1 RING finger and the flanking alpha-helices maintain the orientation of the RING finger with respect to the flanking alpha-helices. The interaction between BRCA1 and BARD1 both increases the ubiquitin ligase activity of BRCA1 and causes the nuclear export sequence (NES), located on the C-terminal helix of the RING domain of both BRCA1 and BARD1, to be buried [13, 32, 33]. The buried NES in the four helix bundle results in nuclear retention of the two proteins. The four helix bundle contains the majority of the interactions between BRCA1 and BARD1, however a few inter-RING interactions may occur as well [13]. As stated above, the RING finger of BRCA1 consists of a small three strand antiparallel β-sheet and a central helix. Two Zn^2+^ atoms stabilize the structure within the RING finger and are coordinated by Zn^2+^ binding loops named Site I and Site II. Site I is made up of four cysteine residues, while Site II contains three cysteine residues and one histidine residue. The Zn^2+^ binding residues are highly conserved and characteristic of RING fingers found in many other proteins. Additionally, the spacing between the Zn^2+^ binding residues is conserved among many RING fingers. Conversely, a central helix is present in some RING fingers, but not all [13].

Ubiquitination of substrates occurs in a three-step process. First, an E1 ubiquitin-activating enzyme activates a ubiquitin (Ub) molecule, which is transferred to an E2 ubiquitin-conjugating enzyme. The E3 brings together the E2 and substrate to complete the ubiquitination process. The human genome encodes ∼40 E2 enzymes, which rely on ∼1000 E3 ubiquitin ligases for their specificity [34]. RING E3 ubiquitin ligases, including BRCA1, act solely as scaffolds by binding to the E2 via the RING finger domain, while the substrate binds to another domain on the E3. This brings the substrate close enough to the E2 to allow for transfer of Ub from the E2 to the substrate. The presence of the E2 ubiquitin-conjugating enzyme, UbcH5, dramatically increases BRCA1/BARD1 ubiquitination activity *in vitro* [28]. NMR structures of BRCA1/BARD1/UbcH5c show that loops of UbcH5c bind to a groove formed by the two Zn^2+^ binding sites and the central helix of the RING finger of BRCA1, and that UbcH5c has no interaction with BARD1 [29]. Several other E2 proteins have been shown to interact with the BRCA1/BARD1 heterodimer in a yeast-two hybrid study [35]. Targets of BRCA1 E3 ligase activity *in vivo* include estrogen receptor-alpha, progesterone receptor, CtIP, and histone protein H2A with resulting alterations in gene activation, DNA repair, and DNA condensation [36–40].

BRCA1 is also subject to autoubiquitination in *in vitro* experiments. Depending on the specific E2 interaction, either mono or poly-autoubiquitination can occur. Additionally, Lys63, Lys48 and Lys6 polyubiquitin chains can be conjugated to BRCA1. Two modes of BRCA1/BARD1 autoubiquitination have been established. “Substrate-specific” monoubiquitination by the E2s UbcH6, Ube2e2, UbcM2, Ube2w and UbcH5 result in the conjugation of a single Ub residue to BRCA1 [35]. “Ubiquitin-specific” E2s, Ubc13, Ube2k and UbcH5 recognize monoubiquitinated BRCA1 and stimulate the conjugation of Lys6, Lys48, and Lys63 polyubiquitin chains to BRCA1 [35]. Thus, different E2 enzymes mediate the mono and polyubiquitination of BRCA1 *in vitro*.

### Cancer-related mutations

Mutation of the cysteine residues that coordinate the Zn^2+^ atoms have been reported as clinically important, indicating that they result in altered function and an increased risk of cancer. Mutation of residues in Site I result in altered folding of the RING domain [13]. A more complete study of Site II residue mutations found altered structure by mass spectrometry and reduced Zn^2+^ binding at Site II [41]. This study reported that BRCA1/BARD1 heterodimerization was not affected by Site II mutations, however a later study by the same group reported that several Site I and Site II mutations caused not only a decrease in ubiquitin ligase activity, but also a decrease in co-immunoprecipitation of BRCA1 and BARD1 [29]. These studies suggest that mutation of Site I and Site II residues may affect BRCA1 ubiquitin ligase activity by either decreasing BRCA1/BARD1 heterodimerization or BRCA1/UbcH5 interaction. Another study has shown that the E3-ubiquitin ligase activity of BRCA1 is inhibited by platinum (Pt)-based alkylating chemotherapeutic drugs [42, 43]. Cisplatin forms adducts through its Pt atom with His117 of BRCA1, causing conformational changes and inhibiting the E3-ubiquitin ligase activity in vitro [43]. Other Pt-based drugs had similar functional effects. Transplatin, carboplatin and oxaliplatin all reduced the E3 ligase activity of BRCA1 at therapeutically relevant concentrations [42]. The large number of RING domain mutations that result in increased risk of breast cancer and the effect of chemotherapeutic drugs on RING domain activity suggest an important role for the RING domain in tumor suppression (but see [30]).

## Exon 11-13

**Figure 3:**
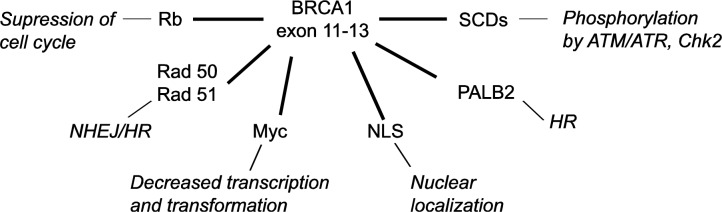
**BRCA1 exons 11-13 have multiple functions**. The amino acids encoded by BRCA1 exons 11-13 have binding domains for several proteins including retinoblastoma (RB), Rad 50, Rad51, c-Myc and PALB2 (a scaffold for BRCA2). BRCA1 exons 11-13 also contain a nuclear localization signal (NLS) and a serine cluster domain (SCD).

Exons 11-13 cover over 65% of the sequence of BRCA1 and encode two nuclear localization sequences (NLS) and binding sites for several proteins including retinoblastoma protein (RB), cMyc, Rad50 and Rad51 (reviewed in [44]). The amino acids encoded by these exons also contain portions of a coiled-coil domain which mediates interactions with PALB2, as well as a portion of a serine containing domain (SCD) that is phosphorylated by ATM (Figure 3). No atomic-level structures have been determined for exons 11-13 of BRCA1. Despite the fact that exons 11-13 contain a large percentage of the clinically relevant mutations, very little is known about the structure or function of this region when compared to the RING or BRCT domains [12]. Interestingly, BRCA1 exon 11-13 binding partners are involved in a wide range of cellular pathways. Myc is a transcription factor for a large number of genes. Rad50, Rad51 and PALB2 are involved in DNA repair. RB controls cell cycle progression. The large number of mutations occurring in this region, many with loss of large portions of sequence, suggests that this region is important for the tumor suppressor function of BRCA1.

### Retinoblastoma protein

The phosphoprotein RB is a well-known tumor suppressor that controls growth by regulating progression through the cell cycle [45]. BRCA1 interacts with the hypo-phosphorylated form of RB *via* BRCA1 exon 11. Specifically, amino acids 304-394 were found to be responsible for binding to the ABC domain of RB [46]. Over-expression of BRCA1 in cells expressing wild type RB causes suppression of cell cycle progression. Deletion of the region of BRCA1 that mediates BRCA1/RB binding inhibits BRCA1-dependent suppression of cell cycle progression [46]. This suggests that the exon 11-mediated interaction between BRCA1 and RB causes cell cycle arrest through actions of RB. This finding also indicates that exon 11 is responsible for BRCA1-dependent cell cycle arrest, and this may also be dependent on the BRCA1/RB interaction.

### Rad50 and Rad51

Rad50 and Rad51 are two proteins involved in DNA repair. Rad50 functions in a complex that includes MreII and Nbs1. This complex is involved in both non-homologous end joining (NHEJ) as well as homologous recombination (HR). An interaction between BRCA1 and Rad50, and therefore with the Rad50/MreII/Nbs1 complex, has been established. This interaction requires BRCA1 exon 11 amino acids 341-748 [47]. BRCA1 recruits the Rad50/MreII/Nbs1 complex to sites of DNA double strand breaks to facilitate DNA repair. BRCA1-null mouse embryonic fibroblast cells exhibit decreased levels of NHEJ activity, which suggest that BRCA1 is involved in the NHEJ process through interaction with the Rad50/MreII/Nbs1 complex. Rad51 is a homologue of the yeast protein RecA and binds to ssDNA, facilitating homologous recombination (HR). BRCA1 is associated with Rad51 during both mitotic and meiotic cells via amino acids 758-1064 [48]. BRCA1 association with Rad50 and Rad51 suggests a role for exon 11 in both NHEJ and HR processes of DNA repair.

### c-Myc

The transcription factor c-Myc also interacts with BRCA1. Reports have indicated that c-Myc promotes transcription of up to 15% of the genome, making it a major hub for transcriptional activation [49]. BRCA1 has two c-Myc binding sites (known as MB1 and MB2). MB1 is located only in exon 11 (a.a. 433-511) while MB2 is located in exons 8-11 (a.a. 175-303) [50]. In SVD-P5 cells co-transformed with c-Myc/Ras, transfection with BRCA1 significantly decreased the ability of these cells to form transformed foci [50]. This suggests that the transformation activity of c-Myc/Ras is inhibited by BRCA1 expression. Additionally, the transcriptional activity of Myc is decreased by BRCA1 [50]. Thus, suppression of the oncogenic activities of c-Myc may account for some of the tumor suppressor activity of BRCA1.

### Nuclear Localization Sequences

Exon 11 contains two nuclear localization sequences (NLS). Amino acids 501-507 (NLS1) and 607-614 (NLS2) are both recognized by importin-α machinery to mediate BRCA1 transport from the cytosol to the nucleus. While both sequences are recognized by importin-alpha, NLS1 is the most critical sequence because mutation of this sequence inhibits all interactions between BRCA1 and importin-alpha [51]. Mutation of the NLS sequences results in altered subcellular localization of BRCA1, with a shift toward cytosolic localization. Clearly, mutations of BRCA1 NLSs causing cytosolic expression of BRCA1 would decrease the tumor suppressor activity of BRCA1 due to the loss of BRCA1's DNA repair activity and subsequent increase in unrepaired mutations and chromosomal abnormalities.

### PALB2

A putative coiled-coil domain spanning exons 11-13 in BRCA1 (a.a. 1364-1437) contains the binding site for PALB2. At this site, PALB2 acts as a scaffold to bring together BRCA1 and BRCA2 to form a complex of the three proteins which is involved in HR during DNA repair. Both BRCA1 and PALB2 contain coiled-coil domains that mediate the interaction of the two proteins. Through modeling of the coiled-coil domain of BRCA1 and PALB2, the interaction sites were mapped to the predicted *a*-face of the PALB2 helix containing Lys14, Leu21, Tyr28, Leu35, and Glu42 and the predicted *a* and *d*-faces of BRCA1 [52]. Mutations in the coiled-coil region of BRCA1 led to the discovery of the PALB2 binding site on BRCA1, since mutations reported in this region (Met1400Val, Leu1407Pro, and Met1411Thr) inhibit interaction between BRCA1 and PALB2 [52].

### Serine Cluster Domain

BRCA1 contains a domain called the serine cluster domain (SCD). A portion of the SCD of BRCA1 is located in exons 11-13, and spans from amino acids 1280-1524. The region has a concentrated amount of putative phosphorylation sites, and is phosphorylated by ATM/ATR kinases *in vitro* and *in vivo*. ATM and ATR are kinases activated by DNA damage. Phosphorylation of BRCA1 causes recruitment of BRCA1 to sites of double strand breaks. SCDs are common in ATM/ATR targets including multiple DNA damage response proteins [53]. Serines 1189, 1457, 1524, and 1542 can all be phosphorylated *in vivo*, while additional serines can be phosphorylated *in vitro* [54]. Mutation of these serine residues are seen clinically, and may affect localization of BRCA1 to sites of DNA damage and DNA damage response function.

## BRCT domain

The BRCA1 C-terminal (BRCT) domain was originally identified in BRCA1, but it is also a conserved domain in multiple other proteins (most being involved in DNA damage repair). BRCT domains can occur as a single BRCT domain, as a tandem repeat (as found in BRCA1), multiple repeats, or fusions between two domains (reviewed in [55]). The BRCA1 BRCT domain mediates phosphoprotein interactions between BRCA1 and proteins phosphorylated by ATM and ATR, two kinases activated by DNA damage (reviewed in [56]). BRCT domains are classified into two categories based on their ability to recognize phosphoproteins. Class-I BRCT domains can recognize phosphoserine (pSer) residues, while Class-II BRCT domains can recognize both pSer and phosphothreonine (pThr) residues. The BRCA1 BRCT domain recognizes the sequence pSer-X-X-Phe in its phosphorylated binding partners, and is therefore a Class-I BRCT domain. Binding partners for the BRCA1 BRCT domain include BACH1, CtIP, and CCDC98/abraxas [57–59]. While the main function of the BRCA1 BRCT domain is modulating interactions between BRCA1 and phosphoproteins, BRCT domains, including the BRCT domain of BRCA1, can also mediate DNA binding and non-phosphoprotein interactions [60].

### Structure-function of the BRCT domain

**Figure 4:**
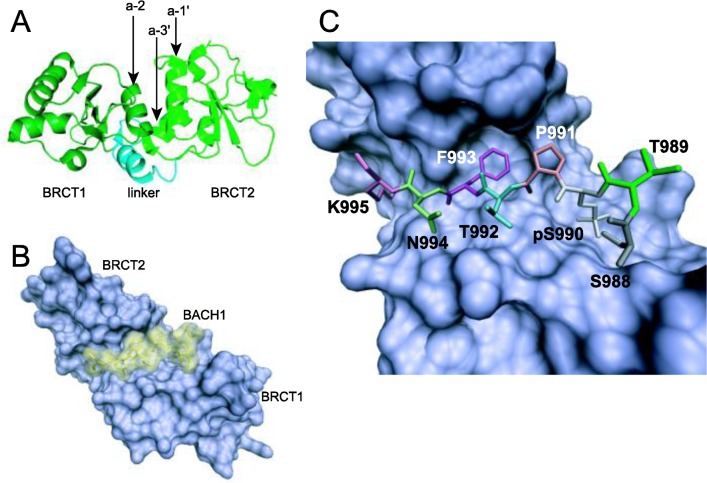
**BRCA1 BRCT tandem repeats recognize phosphoproteins**. A) BRCT1 and BRCT2 pack together in a head-to-tail orientation and are connected by a linker helix. Helix 2 from BRCT1 and helices 1 and 3 from BRCT2 form a hydrophobic core and stabilize the structure. Rendering was generated using POLYVIEW-3D [31]. Structural model is derived from PDB accession number 1T29. B) The cleft between BRCT1 and BRCT2 forms the binding pocket for proteins phosphorylated by ATM and ATR. The BRCA1 BRCT domains are shown in blue, and a fragment of BACH1 is shown in yellow. C) A magnification of the BRCA1 BRCT/BACH1 binding pocket. The consensus sequence for BRCA1 BRCT recognition of phosphoproteins is ^990^pSer-X-X-Phe^993^. The BRCA1-binding region of a phosphopeptide derived from BACH1 is shown. Phospho-Ser990 (pS990) interacts with Ser1655 and Lys1702 of BRCA1, which form a basic pocket. The 3+Phe993 fits into a hydrophobic pocket created by the two BRCT domains (Phe1704, Met1775, Leu1839). Lysine 995 (K995) forms a salt bridge with Asp1840 and Glu1836. Rendering of B and C was generated using Visual Molecular Dynamics (VMD) [62]. Structural model in B and C is derived from PDB accession number 1T15.

Amino acids 1650-1863 of BRCA1 consist of two tandem BRCT repeats connected by a 22 amino acid linker [14]. Each BRCT repeat consists of three α-helices packed around a four strand β-sheet (Figure 4A). The two BRCT repeats interact in a head-to-tail fashion through the interaction between α-helix 2 of BRCT1, and α-helices 1 and 3 of BRCT2 through mainly hydrophobic residues. The architecture of the tandem BRCT allows the BRCA1 BRCT to recognize both a pSer and the 3+aromatic residue in a bipartite manner in two separate recognition pockets in the cleft between BRCT1 and BRCT2 (Figure 4B) [15, 19] (also reviewed in [55]). The pSer residue forms hydrogen bonds with Ser1655 and Lys1702 and the backbone amine group of Gly1656, all within the N-terminal BRCT1 [19]. The 3+phenylalanine residue fits into the hydrophobic core created by the two BRCT repeats, while the main chain backbone of the 3+phenylalanine forms hydrogen bonds with R1699 of α-helix 1 of the N-terminal BRCT domain (Figure 4C) [19]. The size and subsequent rigidity of the hydrophobic core of the interface between the two BRCT repeats dictates the strict consensus sequence required for substrate recognition by the BRCA1 BRCT domain. The consensus sequence pSer-X-X-Phe facilitates recognition of targets such as CtIP, BACH1, and abraxas which are all phosphorylated in response to DNA damage (reviewed in [55]).

The BRCA1 BRCT domain has also been shown to bind directly to DNA double strand breaks (DSB) by electron microscopy [60]. However, the *in vivo* relevance of this interaction is unknown. While structural studies of the interaction between the BRCA1 BRCT domain and double strand breaks have yet to be carried out, models have been developed with predicted interactions between the BRCT of replication factor RFCp140 and DNA double strand breaks. The BRCT domain of RFCp140 recognizes the terminal 5’ phosphate of a 3’ overhanging DNA double strand break, as well as the major groove of the DNA adjacent to the double strand break [61]. It is unknown whether or not the BRCT domain of BRCA1 binds to DNA in a similar manner. BRCT domains have also been shown to interact with some proteins in a phosphorylation-independent manner, however this has been much less studied and not well characterized in BRCA1 (reviewed in [55]).

### BRCT cancer predisposing mutations

Multiple studies have found mutations in the BRCT domain of BRCA1 in breast and/or ovarian cancers [63–65]. Specifically, mutation of hydrophobic residues within the hydrophobic core of the BRCT domain inhibits the ability of BRCA1 to recognize phospholigands [19]. This would suggest that mutation of a residue required for recognition of a substrate would impede the ability of BRCA1 to carry out its role in the DNA damage repair pathway. An interesting example causes BRCA1 to fall into a “similarity trap” [66]. Typically, phosphorylated p53 has a much higher binding affinity for 53BP1 (p53 binding protein 1), than BRCA1. Both 53BP1 and BRCA1 interact with p53 through their tandem BRCT domains, however with different affinities. Two cancer causing mutations in BRCA1, Phe1695Leu and Asp1733Gly cause BRCA1 to bind p53 with similar affinity to 53BP1 [66]. This suggests that these mutations of BRCA1 in the BRCT domain could force BRCA1 into a similarity trap, causing 1) BRCA1 to bind p53 with higher affinity than wild-type BRCA1, and 2) competition for 53BP1 binding to p53. Thus, it is likely that these mutations in the BRCA1 BRCT domain lead to altered p53 function possibly contributing to the cancer phenotype. Another study has shown that cancer causing mutations in other areas of the BRCA1 BRCT domain can alter the backbone structure of the BACH1 binding pocket [67]. This suggests that mutations that affect the BACH1 binding pocket are not limited to just the residues in direct contact with the phosphopeptide. The number of cancer causing mutations in this region suggests that this domain is critical for tumor suppression.

## Clinical Implications

Structural biology has greatly increased our knowledge of BRCA1 structure and function. Of clinical relevance, atomic resolution models have elucidated how BRCA1 missense mutations affect structure (see Table 1), with direct implications for understanding disease pathogenesis. For example, the x-ray structure of the V1809F mutant revealed at the molecular level how this mutation disrupts phosphoprotein binding, indicating a mechanism by which this mutation leads to loss of function and increased cancer risk [15].

Determining the structure of BRCA1 also has implications for the development of future therapeutic treatments. PARP (poly (ADP-ribose) polymerase) inhibitors have been shown to be effective in treating BRCA1-mutated tumors. PARP is activated by DNA single strand breaks (SSBs) to form long, branched ADP-ribose chains that act as scaffolds to recruit other proteins involved in base excision and SSB repair. These proteins then resolve the SSB. PARP inhibition leads to accumulation of SSBs, followed by collapse of replication forks, and finally formation of DSBs [68]. As previously described, BRCA1 is a major component of the homologous repair (HR) pathway that is responsible for resolution of DSBs. PARP inhibition is especially effective in tumors and cell lines lacking homologous repair (HR) activity such as BRCA1-mutated tumors. The RING domain of BRCA1 has been shown to be partly responsible for sensitivity of tumors to the PARP inhibitor olaparib, and this is due to inhibition of BRCA1/BARD1 interaction as well as the BRCA1/E2 ligase interaction [69]. Therefore a rationally designed drug that targets the BRCA1/BARD1 or BRCA1/E2 interface, thus inactivating the HR activity, may sensitize tumors to PARP inhibition. BRCA1 activity is also regulated by cyclin-dependent kinase-1 (Cdk1). Phosphorylation of BRCA1 by Cdk1 promotes the association of BRCA1 to sites of DNA damage [70]. Inhibition of Cdk1 potentiates the sensitivity of cancer cells to PARP inhibitors by reducing BRCA1 recruitment and subsequent repair of DSBs [71]. Thus, drugs indirectly targeting BRCA1 activity also have promise as anti-neoplastic agents when combined with PARP inhibitors.

Drugs targeting the BRCA1 BRCT phosphopeptide binding domain may also have therapeutic potential. The structure of the BRCA1 BRCT domain with phosphopeptides derived from both CtIP and BACH1 have been determined (See Table 1) [16], [18]. The BRCA1/CtIP interaction controls the G2/M transition checkpoint, while the BRCA1/BACH1 interaction controls the G2 accumulation checkpoint [58]. As suggested by Varma *et. al*., a rationally designed drug that inhibits phosphopeptide binding to BRCA1 would be expected to disrupt G2 cell cycle arrest, leading to genomic instability, apoptosis, and increased susceptibility to chemotherapeutic agents [16]. Thus, as the examples above demonstrate, structural studies of BRCA1 are essential for understanding disease pathogenesis and the discovery of novel therapeutics.

## Conclusions

The high rate of mutations in specific domains of BRCA1 suggests that these domains are critical for its tumor suppressor activity. Studies into the structure and function of BRCA1 have greatly increased our understanding of the molecular mechanisms through which mutations cause predisposition to breast and ovarian cancers. However, more studies are needed to fully understand the molecular function of BRCA1. While structural studies of the RING and BRCT domains have greatly increased our knowledge of BRCA1, these domains only cover 17% of the BRCA1 primary sequence. Structural studies of exons 11-13 as well as the rest of the BRCA1 protein will be necessary to elucidate the molecular basis by which mutations in these domains lead to cancer predisposition.
